# Non-destructive geographical traceability of sea cucumber (*Apostichopus japonicus*) using near infrared spectroscopy combined with chemometric methods

**DOI:** 10.1098/rsos.170714

**Published:** 2018-01-17

**Authors:** Xiuhan Guo, Rui Cai, Shisheng Wang, Bo Tang, Yueqing Li, Weijie Zhao

**Affiliations:** 1 School of Pharmaceutical Science and Technology, Dalian University of Technology, No.2 Linggong Road, Hi-Tech Industry Zone District, Dalian 116023, People's Republic of China; 2 State Key Laboratory of Fine Chemicals, Dalian University of Technology, No.2 Linggong Road, Hi-Tech Industry Zone District, Dalian 116023, People's Republic of China; 3 School of Chemical Engineering, Dalian University of Technology, No.2 Linggong Road, Hi-Tech Industry Zone District, Dalian 116023, People's Republic of China

**Keywords:** *Apostichopus japonicus*, geographical origin, traceability, total fat content, near infrared spectroscopy, chemometrics

## Abstract

Sea cucumber is the major tonic seafood worldwide, and geographical origin traceability is an important part of its quality and safety control. In this work, a non-destructive method for origin traceability of sea cucumber (*Apostichopus japonicus*) from northern China Sea and East China Sea using near infrared spectroscopy (NIRS) and multivariate analysis methods was proposed. Total fat contents of 189 fresh sea cucumber samples were determined and partial least-squares (PLS) regression was used to establish the quantitative NIRS model. The ordered predictor selection algorithm was performed to select feasible wavelength regions for the construction of PLS and identification models. The identification model was developed by principal component analysis combined with Mahalanobis distance and scaling to the first range algorithms. In the test set of the optimum PLS models, the root mean square error of prediction was 0.45, and correlation coefficient was 0.90. The correct classification rates of 100% were obtained in both identification calibration model and test model. The overall results indicated that NIRS method combined with chemometric analysis was a suitable tool for origin traceability and identification of fresh sea cucumber samples from nine origins in China.

## 1. Introduction

Sea cucumber is a typical bottom-dwelling animal which pertains to the phylum Echinodermata (Aspidochirotida: Holothuroidea). *Apostichopus japonicus*, a species of sea cucumber mainly distributed in northern waters of the western Pacific, has a long history of dietary and medical uses in Asian countries, especially in China, Japan and Korea, owing to its various healthcare functions, including anti-tumour [1], immunoregulation [2], anti-atherosclerosis, antioxidant and antiapoptotic effects [3]. In China, there are more than 20 species of edible sea cucumbers widely distributed in the areas of the Yellow Sea, the Bohai Sea and the East Sea. Among them, the Yellow Sea and the Bohai Sea are primary producing areas famous for the high quality of sea cucumber, such as *A. japonicus*, which is one of the most valuable species and possesses large market share in China.

Since sea cucumbers of the same species growing in different sea regions have different qualities and medical applications, and they are easily confused, difficult to be identified by sensory methods (sight, smell, taste, etc.), the origin of *A. japonicus* is considered to be a main factor influencing the price and consumer preference. For making exorbitant profits, many tortious acts such as confusing origins of products, selling seconds at best quality prices and adulteration are increased noticeably, and the resulting unscrupulous competitions have damaged consumer benefits and the reputation of geographical indication sea cucumbers. With regard to these severe food safety problems, the urgent countermeasure is to establish rapid and feasible methods for source identification and traceability of sea cucumber.

Many research works have been carried out for origin traceability and identification of sea cucumber. Multi-element analysis with pattern recognition techniques [4] and the stable isotope ratios and fatty acid (FA) profiles analysis [5] have been used to evaluate the applicability in the origin traceability of sea cucumbers from different sampling points in China Sea. Diffuse reflectance mid-infrared Fourier transform spectroscopy [6] and species-specific polymerase chain reaction method [7] have been used for rapid identification of dried sea cucumber products from different geographical areas. These traditional methods are precise and reliable; however, most of them are often time-consuming and sophisticated, or require complex pre-processing [8,9]. Hence, rapid and accurate control methods are usually preferred, and one of the most frequently chosen and proposed method is near infrared spectroscopy (NIRS) technology. NIRS combined with chemometric methods is time-saving, cost-efficient and without the need of tedious sample preparation, and it has shown a good adaptability in authentication [10,11], origin traceability, discrimination on food safety problems [12–15], qualitative and quantitative analysis of natural products [16,17], pharmaceuticals [18–20], as well as monitoring and controlling product quality in agri-food processing [21–23]. In recent years, many studies on the quality control of seafood, distinction of geographical origin and content determination by NIRS have been reported [24–27]. However, the identification of the geographical origins of fresh sea cucumber using NIRS has not been reported so far.

Chemometric methods offer more possibilities to analyse a large amount of data obtained by NIRS. One of the main issues of NIRS model establishment is to select the most informative wavelength regions. To extract the effective information and to remove irrelevant features, various methods have been developed, and one of these approaches is the ordered predictor selection (OPS) algorithm [28]. OPS has been used to select wavelengths for NIR analysis of coffee [29] and chemicals [30]. In our preliminary experiment, the result reveals the difficulty of choosing satisfactory wavelengths for identification of fresh sea cucumber. In this study, we intend to take the total FA content as the index for the wavelength region selection for identification model establishment by OPS, because the total fat contents and profile have been successfully used as biomarkers to confirm and trace the geographical origins of *A. japonicus* in some reports [5,31]. The principal component analysis combined with Mahalanobis distance (PCA-MD) algorithm and the scaling to the first range algorithm [32] have been successfully applied in our previous work for origin traceability identification of Chinese propolis [12] and *Corydalis tuber* [33], respectively. In view of the power of these methods in identifying the origins of natural products with complex components, they were adopted as identification chemometric methods in this study.

To supply a new access to develop rational quality standards for improving the quality control of various sea cucumber (*A. japonicus*) origins, the major objective of this study was to develop a rapid, cheap and non-destructive method for identification of fresh *A. japonicus* from nine sampling locations in northern China Sea and East China Sea by using NIRS. The detailed aims of this study were to (i) select suitable wavelengths which could give best results for origin identification of *A. japonicus* by using OPS algorithm, (ii) establish NIRS identification model with PCA-MD and scaling to the first range algorithms to identify origin of *A. japonicus* and (iii) develop NIRS quantitative model for contents of total fat in *A. japonicus* with partial least-squares (PLS) regression.

## 2. Material and methods

### 2.1. Apparatus and reagents

The NIR spectra of *A. japonicus* were collected using an MPA™ Fourier transform near infrared spectrometer (Bruker Optics, Germany), which was equipped with reflectance fibre-optic probe, indium-gallium-arsenide (InGaAs) and lead sulfide (PbS) detector. Data analysis was performed by Matlab 7.1 software and OPUS 6.5 software from Bruker Optics. OPS algorithm was performed using OPS_Toolbox package, which was available at www.deq.ufv.br/chemometrics.

Methanol and chloroform (Merck, Germany) were of HPLC grade. The other chemical reagents used in the experiments were bought from Energy Chemical Corp. (Shanghai, China). Ultrapure water was produced by a Milli-Q water system (Millipore Corp., MA, USA). N_2_ (99.999% purity) and He (99.999% purity) were provided by Junfeng Gas Co. (Dalian, China).

### 2.2. Sample collection and preparation

**Figure 1.**
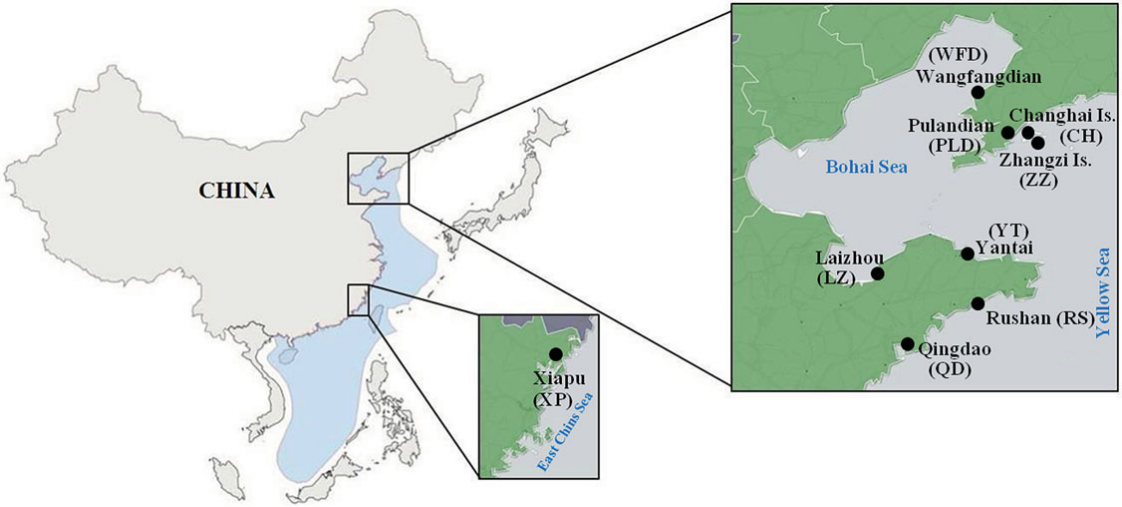
Sampling locations of *A. japonicus* from the Bohai Sea, Yellow Sea and East China Sea.

We collected 189 *A. japonicus* samples (3-year-old adult individuals) from nine habitats of three water environments, including main producing areas of *A. japonicus* of the northern China Sea and the East China Sea. All the samples were grown in 30 m depth deep sea and were not artificially fed. The detailed information is listed in table 1 and locations of sampling sites are shown in figure 1. All samples were dissected as soon as they were caught, and their digestive tracts were cleared away immediately. The body walls were kept in a freezer at −20°C during sampling and 2-day transport to laboratory. Then, each sample (body wall) was transected into two even parts (Part-A and Part-A′). Part-A was used without any pretreatment prior to spectrum acquisition, and Part-A′ was used for determination of total fat contents. Completely frozen Part-A′ was freeze-dried at −50°C for 48 h, and the dried samples were pulverized with a mortar and pestle, passed through an 80-mesh sieve, and then stored under dry conditions.

**Table 1.** Information of sea cucumber *A. japonicus* samples collected from different origins of China Sea.

water environment	origin	sample codes	latitude--longitude pairs	number	sampling time	average length (cm)	average weight (g)
Yellow Sea	Changhai	CH	39°15′42.8′′ N,	22	Nov 2015	15.3 ± 2.9	114 ± 12.3
			122°36′1′′ E				
Bohai Sea	Wanfangdian	WFD	39°46′3′′ N,	17	Nov 2015	16.0 ± 1.6	115 ± 11.5
			121°29′31′′ E				
Yellow Sea	Zhangzi Island	ZZ	39°1′8′′ N,	20	Nov 2015	16.3 ± 1.8	109 ± 20.7
			122°45′34′′ E				
Yellow Sea	Pulandian	PLD	39°26′54′′ N,	22	Nov 2015	16.3 ± 2.0	115 ± 12.7
			122°22′32′′ E				
Yellow Sea	Qingdao	QD	35°55′57′′ N,	20	Dec 2015	16.0 ± 1.9	110 ± 10.6
			120°21′44′′ E				
Yellow Sea	Rushan	RS	36°46′46′′ N,	21	Dec 2015	15.7 ± 3.4	115 ± 12.7
			121°34′59′′ E				
Yellow Sea	Yantai	YT	37°26′54′′ N,	30	Dec 2015	16.1 ± 1.2	115 ± 12.0
			121°26′19′′ E				
Bohai Sea	Laizhou	LZ	37°15′34′′ N,	17	Dec 2015	15.9 ± 2.1	112 ± 16.7
			119°52′22′′ E				
East China Sea	Xiapu	XP	26°52′54′′ N,	20	Dec 2015	16.2 ± 2.0	116 ± 10.2
			120°5′47′′ E				

### 2.3. Spectra collection

To minimize the deviation caused by human factors, each *A. japonicus* sample was placed into a test tube with 1.2 cm inner diameter, and the reflectance fibre-optic probe with 1.1 cm diameter was plugged into the tube and ‘fixed’ tightly on the surface of the sample to record NIR spectra. Each spectrum was measured in diffuse reflection mode in the range of 800−2500 nm with a 2 nm spectral resolution and an average of 64 scans. A background spectrum was recorded prior to analysing each sample. The temperature was kept around 25°C and the humidity was kept constant. The original spectrum for establishing NIR model was the average of three spectra recorded in different sites of each sample.

### 2.4. Quantitative analysis of total fat contents in *Apostichopus japonicus*

FA analyses of the samples were carried out according to Zhang *et al*. [5] and Xu *et al*. [31]. The Folch method of lipid extraction [34] was used in this study for total lipid extraction from *A. japonicus*. The powdered sample (0.5 g) was added to 10 ml of chloroform–methanol solution (2:1, v/v), and extracted by mechanical shaking for 30 min at 37°C, and extracted overnight, and then filtered. The extract was washed with 1/5 vol of 0.88% NaCl solution and left to stand for separation. The chloroform layer was collected, dried by anhydrous sodium sulfate and then concentrated under a nitrogen stream to obtain the total lipids fraction.

A 20 mg sample of the total lipids was dissolved in 1 ml of 1% sulfuric acid--methanol solution in a 60°C water bath for 60 min to carry out methylation and then cooled. Subsequently, 1 ml of *n*-hexane was added, and the mixture was sonicated for 30 s and then left to stand. The supernatant was reserved and analysed by an Agilent 7890A/7000B triple quadrupole gas chromatography/mass spectrometry (GC/MS) instrument equipped with a capillary column HP-5 (30 m × 0.25 mm ID coated with 0.25 µm film thickness; Agilent, Santa Clara, CA). The programme of temperature was as follows: 70°C for 3 min, held at 220°C for 33 min after increasing at a rate of 3°C min^−1^. Temperatures of the injector and detector were set at 220 and 280°C, respectively. The injection volume was 1.0 µl. The carrier gas was He (99.999% purity), introduced at a flow rate of 1.2 ml min^−1^ in constant-flow mode. For MS measurement, the transfer-line temperature was 250°C, and the ion-source temperature was 230°C. Ionization of samples was carried out in electron ionization mode at electron energy of 70 eV. The GC retention time and the MS spectra of samples were, respectively, compared against the GC retention time of Supelco 37 Component FAME Mix (Supelco, Bellfonte, PA) and the MS spectra of a standard library (NIST2010) to determine the types of FA. Total fat content value was defined as the sum of the identified individual FA value and reported as mean values ± standard deviation, and analysed by one-way analysis of variance (ANOVA). Significant differences were determined by using Tukey's honestly significant difference (HSD) *post hoc* test (SPSS Statistics 20.0).

### 2.5. Chemometric processing

To establish a feasible NIRS identification model and PLS model, pre-processing methods were applied to eliminate unnecessary physical information and magnify relevant variations in original spectra. Different pre-processing methods were compared in accordance with their predictive performance, including the first derivative (FD), second derivative (SD), standard normal variate (SNV), [35] multiplicative scatter correction (MSC), [36] vector normalization (VN; firstly, the spectra are centred; then, the sum of all squares of all Y-values is calculated, and the respective spectrum is divided by the square root of this sum; the vector norm of the resulting spectrum is 1) [36], and SD-VN. To avoid enhancing the noise by SD, spectra were smoothed at first by Savizky–Golay (S.G) smoothing algorithm [37].

In this study, visible and short wavelength region (800−1000 nm) was excluded due to the high level of noise caused by instruments. The region of 1800−2500 nm was not applied on account of the absorption spectra were truncated as small amount of low energy light, which could not pass through the samples, meaning absorption spectra above 1850 nm showed stray light [38,39]. The OPS algorithm was applied to select suitable wavelength regions for PLS regression of total FA contents. Then the selected wavelength regions were used to build identification model. The NIR spectra were organized into a matrix format ***X***(*m* × *n*), and matrix ***X*** was transformed into matrix ***X****_p_* by using pre-processing method. Total FA content values were arranged as the dependent variables (***y***), and the pre-processed spectra (matrix ***X****_p_*) of *A. japonicus* samples were used as the independent variables to develop PLS regression model. To validate PLS model, leave-one-out cross validation was used, and the dataset was split as follows: 148 samples were randomly selected to be calibration set, and the remaining 41 samples were arranged in test set.

A ‘two-step’ NIRS identification model (Step I tandem Step II model) was established for classifying the origins of *A. japonicus* samples. The 144 samples were used to establish the calibration model, and the remaining 45 samples (five for each origin) were used for validation. One hundred and forty four samples were classified by Step I model at first, and the unidentified spectra were subsequently entered into the tandem Step II model for further classification. The optimum factors for model establishment of each step (pre-processing method, wavelength regions and identification chemometric method) were selected, respectively, that would enhance the predictive ability of NIRS model.

PCA-MD algorithm and scaling to the first range algorithm were used in Step I and Step II models as the identification chemometric methods, respectively. The detailed formulae could be found in [33]. Selectivity (*S*) was used to evaluate the discriminating ability of identification model, which had been successfully used in our previous work [33,40,41]. *S *> 1 means the classes can be completely separated; *S *= 1 means the classes are in contact; and S < 1 means the classes are overlapping. The larger the *S* value is, the better the discrimination result is, and the more accurate the prediction model is. *S* is calculated by formula (2.1), where *D* denotes the distance from the centre of one category to another. The distance between the unknown spectra and the average spectrum in calibration set (Hit) is calculated, and the threshold for each category (*D*_T_) is calculated by formula (2.2), where *S*_Dev_ is the standard deviation of Hit; *Q* is a coefficient, and 0.25 is usually selected [12].

2.1 $$S=\frac{D}{D_{T1}+D_{T2}}$$

and

2.2 $$D_{T}=\mathrm{Max}(\mathrm{Hit})+{\text{QS}}_{\mathrm{Dev}}.$$

## 3. Results and discussion

### 3.1. Quantitative analysis of total fat contents by gas chromatography/mass spectrometry

According to the method described in §2.4, the determination of total fat contents of *A. japonicus* body wall from nine origins by GC/MS, and the results of ANOVA and Tukey's HSD *post hoc* test are listed in table 2, and those values were used to establish NIRS quantitative model as the standard values. The total fat contents reveal significant differences between *A. japonicus* from different origins. Samples from LZ showed statistically higher levels of total fat contents compared with those of samples from other origins. By contrast, samples from XP showed lower levels of total fat contents.

**Table 2.** The value of total fat content in the body wall of *A. japonicus* from nine origins in China. XP, Xiapu; LZ, Laizhou; YT, Yantai; RS, Rushan; QD, Qingdao; WFD, Wanfangdian; PLD, Pulandian; CH, Changhai; ZZ, Zhangzi Island.

Origin	XP	QD	RS	YT	LZ	WFD	CH	PLD	ZZ
*Σ*F^c^ (%)	4.63 ± 0.38^b^	4.96 ± 0.92^ab^	5.01 ± 0.73^ab^	5.13 ± 1.35^ab^	5.96 ± 2.34^a^	5.84 ± 2.05^ab^	5.66 ± 0.53^ab^	5.76 ± 0.92^ab^	5.33 ± 1.05^ab^

^a,b^Difference of total fat contents is significant (*p* ≤ 0.05).

^c^Total fat content was reported as mean values ± standard deviation.

### 3.2. Results of partial least-squares model

**Figure 2.**
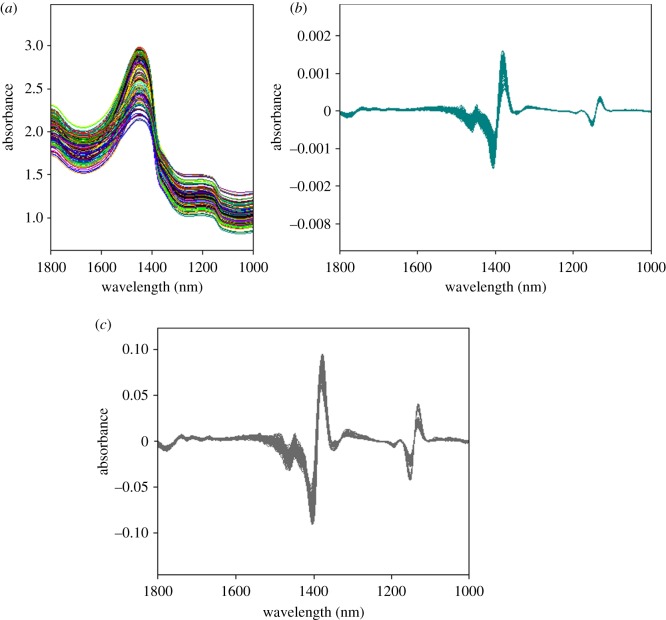
Original (*a*), second derivative after Savitsky–Golay smoothing with a window size of 17 points pre-processed (*b*) and second derivative coupled vector normalization pre-processed (*c*) NIR diffuse reflectance spectra of *A. japonicus* samples.

The OPS algorithm was conducted as follows. The 189 original spectra of *A. japonicus* samples were organized into a format matrix (189 × 700). After the pre-processing investigation, S.G smoothing with 17 spectral points before second derivative was chosen for quantitative model due to the lowest root mean square error of cross-validation (RMSECV) and highest correlation coefficient of cross-validation (*R*^2^) of calibration model. The equation of RMSECV, the root mean square error of prediction (RMSEP) and *R*^2^ could be found in [42]. The original and pre-processed spectra are shown in figure 2.

The number of latent variables (*l*) of PLS model was also determined according to RMSECV and *R*^2^ values. The results of total FA content PLS models achieved by using different pre-processing methods with optimum parameters are listed in table 3. When 17 points smoothing and SD was adopted, *l* equals 8, the lowest RMSECV (0.31) and highest *R*^2^ (0.93) could be obtained.

**Table 3.** The statistics of PLS models of total fat contents with different pre-processing methods. S.G(9.0)-SD, Savizky–Golay smoothing (17 points) before second derivative; FD, first derivative; SD, second derivative; SNV, standard normal variate; MSC, multiplicative scatter correction; VN, vector normalization; *l*, the number of latent variables; RMSECV, the root mean square error of cross-validation; RMSEP, the root mean square error of prediction; *R*^2^, correlation coefficient.

pre-processed method	*l*	RMSECV	RMSEP	*R*^2^ (calibration)	*R*^2^ (test)
S.G(17.0)-SD	8	0.31	0.45	0.93	0.90
SNV	9	0.91	0.99	0.74	0.62
MSC	7	1.87	2.01	0.45	0.40
VN	9	1.09	1.89	0.44	0.37
SD + VN	8	0.31	0.53	0.90	0.81
FD	9	1.23	1.76	0.71	0.56

After *l* value was investigated to build the optimal regression vector used in OPS, 30 regions from 700 absorbance variables were selected to build PLS model of total FA content. The detailed information of selected wavelength regions and relevant absorption bands of functional groups are listed in table 4. The selected regions were mainly associated with the vibration of functional groups of FAs.

**Table 4.** Wavelength regions selected by OPS algorithm for establishing PLS regression model of total fat contents and identification model.

region selected	selected wavelength regions (nm)	vibrational modes^c^	functional groups
1	1145–1208^a^	second overtone of C–H	CH_2_ CH_3_
2	1210–1223^a^^,^^b^	second overtone of C–H	CH CH_2_
3	1225–1246^a^	second overtone of C–H	CH
4	1287–1295^a^	first overtone of C–H combination bands	CH_3_
6	1300–1310^a^^,^^b^	first overtone of C–H combination bands	CH_3_
7	1321–1346^a^^,^^b^	first overtone of C–H combination bands	CH_3_
8	1352–1364^a^	first overtone of C–H combination bands	CH_3_
9	1368–1378^a^	first overtone of C–H combination bands	CH_2_ CH_3_ ArOH
10	1381–1385^a^^,^^b^	first overtone of C–H combination bands	CH_2_ CH_3_ ArOH
11	1386–1390^b^	first overtone of C–H combination bands	CH_2_ CH_3_ ArOH
12	1391–1393^a^	first overtone of C–H combination bands	CH_2_ CH_3_ ArOH
13	1394–1400^a^^,^^b^	first overtone of C–H combination bands	CH_2_ CH_3_ ArOH ROH
14	1402–1410^a^^,^^b^	first overtone of C–H combination bands	CH_2_ CH_3_ ArOH ROH
15	1412–1418^b^	first overtone of C–H combination bands	CH_2_ CH_3_ ArOH ROH
16	1420–1422^a^	first overtone of C–H combination bands	CH CH_2_ ROH
17	1450–1452^b^	first overtone of O–H	CH ROH CONH_2_ CONHR
18	1452–1455^a^^,^^b^	first overtone of O–H	CH ROH CONH_2_ CONHR
19	1458–1462^a^^,^^b^	first overtone of O–H	ROH CONH_2_ CONHR
20	1464–1473^a^^,^^b^	first overtone of O–H	ROH CONH_2_ CONHR
21	1532–1538^a^^,^^b^	first overtone of N–H	RNH_2_
22	1540–1566^a^^,^^b^	first overtone of N–H	RNH_2_
23	1568–1602^a^	first overtone of N–H	RNH_2_
24	1604–1622^b^	first overtone of C–H	ArCH
25	1624–1645^a^^,^^b^	first overtone of C–H	ArCH
26	1648–1651^a^^,^^b^	first overtone of C–H	ArCH CH_3_
27	1654–1660^a^^,^^b^	first overtone of C–H	CH_3_
28	1665–1670^a^^,^^b^	first overtone of C–H	CH_2_ CH_3_
29	1674–1680^a^^,^^b^	first overtone of C–H	CH CH_2_ CH_3_
30	1688–1690^a^^,^^b^	first overtone of C–H	CH CH_2_ CH_3_
31	1698–1700^a^^,^^b^	first overtone of C–H	CH CH_2_ CH_3_
32	1712–1734^a^^,^^b^	first overtone of C–H	CH CH_2_
33	1734–1738^b^	first overtone of C–H and S–H	CH CH_2_ SH
34	1740–1750^a^	first overtone of C–H and S–H	CH CH_2_ SH
35	1750–1758^a^^,^^b^	first overtone of C–H	CH
36	1760–1770^a^	first overtone of C–H	CH

^a^Wavelength regions for establishing Step I calibration model.

^b^Wavelength regions for establishing Step II calibration model.

^c^The spectral band assignments were referred to in [29,32,42,43].

**Figure 3.**
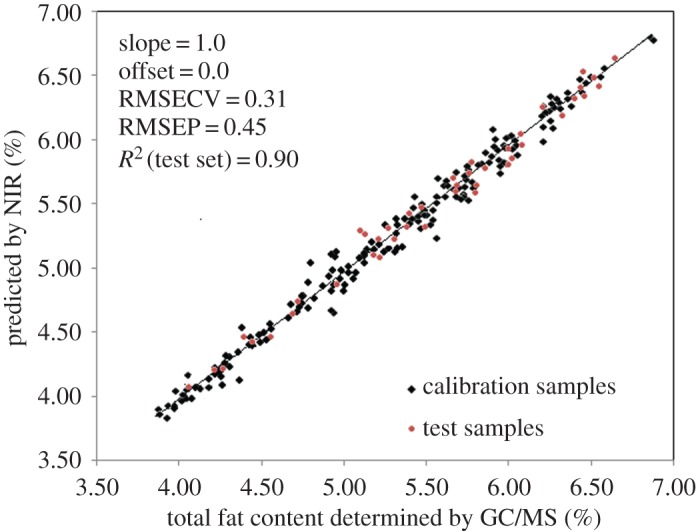
Correlation between the NIRS predicted values and the reference values of total fat in *A. japonicus* samples by PLS in Step I quantitative model (148 samples in calibration set, in black; 41 samples in test set, in red).

The above optimized PLS model was used to predict the total fat contents of 41 samples in the test set, and the scatter plots in figure 3 show the correlation between NIR prediction value and reference GC/MS measurement for fat contents by optimized PLS model. The calibration and prediction data revealed good correlation with reference measurement data in figure 3 where many data points fell on or were close to the unity line.

### 3.3. Results of classification model

**Figure 4.**
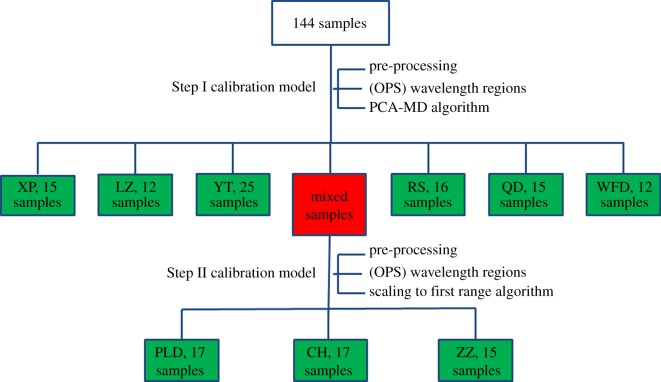
The flow chart of procedures for the ‘two-step’ identification model establishment. Samples in green blocks represent unambiguous classified and samples in red block represent ambiguous classified. XP, Xiapu; LZ, Laizhou; YT, Yantai; RS, Rushan; QD, Qingdao; WFD, Wanfangdian; PLD, Pulandian; CH, Changhai; ZZ, Zhangzi Island.

**Figure 5.**
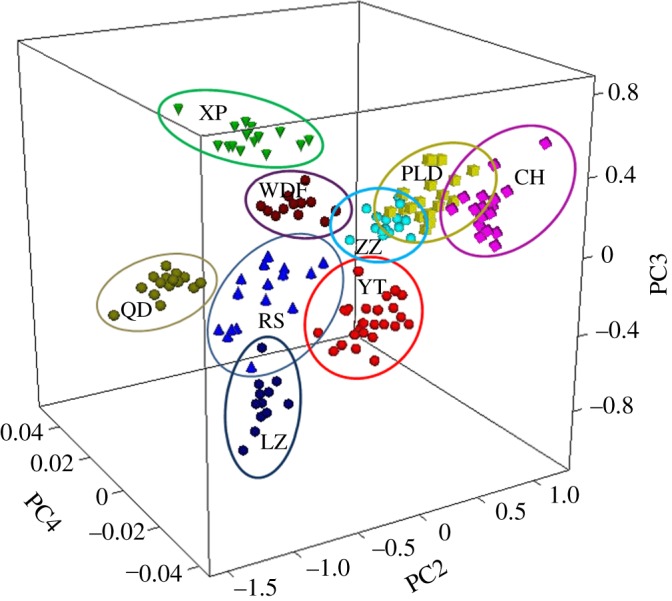
Score plots with three principal components (PCs) for Step I calibration set samples from nine origins including Changhai (CH), Wafangdian (WFD), ZhangziIsland (ZZ), Pulandian (PLD), Qingdao (QD), Rushan (RS), Yantai (YT), Laizhou (LZ), Xiapu (XP), which were obtained by second derivative after Savitsky–Golay smoothing with a window size of 17 points pre-processed.

The flow chart of procedures for the ‘two-step’ identification model establishment is shown in figure 4. The spectral band assignments (in table 4) were presented in [29,32,42,43]. The 30 wavelength regions selected by OPS for PLS model of total fat contents were also used for establishment of Step I identification model. S.G smoothing with 17 points before SD was applied as a pre-processed method, and PCA-MD method was performed on selected wavelength regions (in table 4, with superscript a). After distinguishing by Step I model, 49 samples (red block in figure 4) entered into the tandem Step II for further identification due to the ambiguous classification. The detailed discrimination results (values of *S*) of Step I model are listed in table 5. In table 5, the samples listed in column ‘Group_stepI_ or Group_stepII_’ were the ones that got the minimum difference with the samples in column ‘Group’ (minimum *S* value is obtained), and these minimum *S* values are listed in column ‘*S*_I_ or *S*_II_’. Taking XP samples in the first line (column Group) as an example, after the Step I classification, the ones that got the minimum difference with them were the WFD samples (column Group_stepI_), and the *S* value of Step I was 2.48 (*S*_I_ > 1), which indicates that they can be completely separated, not necessary for the Step II classification. But as to the CH samples in the seventh line, after the Step I classification, the ones that got the minimum difference with them were the ZZ samples (column Group_stepI_), and the *S* value of them was 0.51 (*S*_I_ < 1), which indicates that they have not been separated (overlapping), therefore they needed to go through the Step II classification, and luckily the *S* value of Step II is 1.10 (*S*_II_ > 1), which indicates that they have been successfully separated into the correct clusters. The largest *S*_I_ value was obtained from XP (2.48), and the *S*_I_ value of each group in Step I model was larger than 1.06, except *S*_I_ values of CH (0.51), PLD (0.04) and ZZ (0.04), which indicated significant differences among *A. japonicus* samples from XP, WFD, QD, YT, RS and LZ origins. The results could also be seen from figure 5, where the three-dimensional principal component space provided an overview of the ability of the NIRS identification model to classify *A. japonicus* from nine origins in China Sea. The best discrimination was observed on the score plots in three-dimensional space represented by PC2 (10.3%), PC3 (2.4%) and PC4 (0.3%) vectors. Though the variance contribution rate of PC1 was up to 83.4%, PC1 was not adopted in model construction due to its ambiguous classification. It was speculated that PC1 contained main chemical information of the samples rather than different information between samples. As shown in figure 5, sea cucumber samples from nine different origins were classified into seven concentrated districts. The XP samples could be completely separated from samples of other origins, and the good classification result of samples from XP could be attributed to the significantly different water environments and food sources between the East China Sea and the northern China Sea. On the contrary, the distributions of samples from PLD, ZZ and CH were partially overlapped, which was coincident with their adjacent geographical locations in Yellow Sea, resulting in the similarity in water environments and food sources.

**Table 5.** The results of identification model. *S*_I_: *S* value of Step I model; *S*_II_: *S* value of Step II model; *S *> 1: the classes are completely separated; *S *= 1: the classes are in contact; *S *< 1: the classes are overlapping.

Group	Group_StepI_	*S*_I_	Group_StepII_	*S*_II_
XP	WFD	2.48	—	—
QD	RS	1.06	—	—
RS	QD	1.06	—	—
YT	RS	1.30	—	—
LZ	RS	2.21	—	—
WFD	PLD	2.05	—	—
CH	ZZ	0.51	ZZ	1.10
PLD	ZZ	0.04	ZZ	1.31
ZZ	PLD	0.04	PLD	1.31

The 49 samples (red block in figure 4) that were not successfully classified by Step I model were subsequently separated by the tandem Step II model. SD-VN was used as the pre-processing method, and the scaling to the first range algorithm was performed on 25 wavelength regions selected by OPS (with superscript b, listed in table 4). Finally, *S*_II_ for CH, PLD and ZZ were all larger than 1.10, which indicated that *A. japonicus* samples from CH, PLD and ZZ origins could be properly classified. And the overall results of Step II are shown in table 5.

The 45 samples in the validation set were classified using the established models, and none of them was identified as the wrong class. The overall results indicated that, though the sea cucumber samples investigated in this work belonged to the same species, different geographical regions and water environments would lead to discriminative constituents which revealed discrepant spectral information of samples, and NIRS technique combined with chemometric analysis was a feasible tool to correctly classify *A. japonicus* samples from nine origins in China Sea.

## 4. Conclusion

In this work, the origin traceability and identification method developed by NIRS combined with chemometric analysis provides a satisfactory predictive ability for *A. japonicus* samples from nine origins in China. The correct classification rate by means of the classification model reached to 100%. Moreover, the established PLS model was suitable for the determination of total fat contents of sea cucumber. It is worth mentioning that the NIRS method does not require any complex pre-processing of samples, and the utilization of reflectance fibre-optic probe makes it possible for *in situ* online detection of wild sea cucumbers. Nonetheless, before the NIRS method can be practically applied to origin traceability and identification in sea cucumber industry, more extensive samples are needed to improve the accuracy and applicability of the identification models.

## Data accessibility

Our data are deposited at Dryad (http://dx.doi.org/10.5061/dryad.n1066) [44].
